# Global identification of microRNAs associated with chlorantraniliprole resistance in diamondback moth *Plutella xylostella* (L.)

**DOI:** 10.1038/srep40713

**Published:** 2017-01-18

**Authors:** Bin Zhu, Xiuxia Li, Ying Liu, Xiwu Gao, Pei Liang

**Affiliations:** 1 Department of Entomology, China Agricultural University, Beijing, 100193, P. R. China

## Abstract

The diamondback moth (DBM), *Plutella xylostella* (L.), is one of the most serious cruciferous pests and has developed high resistance to most insecticides, including chlorantraniliprole. Previous studies have reported several protein-coding genes that involved in chlorantraniliprole resistance, but research on resistance mechanisms at the post-transcription level is still limited. In this study, a global screen of microRNAs (miRNAs) associated with chlorantraniliprole resistance in *P. xylostella* was performed. The small RNA libraries for a susceptible (CHS) and two chlorantraniliprole resistant strains (CHR, ZZ) were constructed and sequenced, and a total of 199 known and 30 novel miRNAs were identified. Among them, 23 miRNAs were differentially expressed between CHR and CHS, and 90 miRNAs were differentially expressed between ZZ and CHS, of which 11 differentially expressed miRNAs were identified in both CHR and ZZ. Using miRanda and RNAhybrid, a total of 1,411 target mRNAs from 102 differentially expressed miRNAs were predicted, including mRNAs in several groups of detoxification enzymes. The expression of several differentially expressed miRNAs and their potential targets was validated by qRT-PCR. The results may provide important clues for further study of the mechanisms of miRNA-mediated chlorantraniliprole resistance in DBM and other target insects.

The diamondback moth (DBM), *Plutella xylostella* (L.) (Lepidoptera: Plutellidae), is a major pest of cruciferous vegetables and is known to cause serious losses in agricultural production. The global control and damage costs for this insect pest are estimated at 4–5 billion dollars per year^1^. Due to the long-term use of chemical control coupled with the intensive and irrational use of insecticides, *P. xylostella* has developed resistance to various types of insecticides and has become one of the most resistant pests in the world^2^.

Chlorantraniliprole is a type of anthranilic diamide insecticide with a unique mode of action that activates the muscle ryanodine receptor (RyR)^3^. Because of this novel mode of action, chlorantraniliprole is very effective in controlling several orders of insects, especially lepidopteran pests, and shows no cross-resistance to other commonly used insecticides^3^. However, this insecticide has been applied worldwide since it came on the market, and in recent years, *P. xylostella* has developed high levels of resistance to chlorantraniliprole in many countries, including China^4^,^5^,^6^,^7^.

At present, the research on the mechanisms of chlorantraniliprole resistance in insects is mainly focused on target resistance and detoxification metabolisms. The point mutations in *P. xylostella* RyR^8^,^9^,^10^ and the increased activity of detoxification enzymes including cytochrome P450 monooxygenase (P450), carboxylesterase (CarE) and glutathione S-transferase (GSTs)^11^,^12^ have been demonstrated to be responsible for chlorantraniliprole resistance. However, knowledge of the regulation mechanisms of these genes is relatively limited. Most recently, two miRNAs (miR-7a and miR-8519) were found to be involved in chlorantraniliprole resistance through the up-regulation of RyR expression in *P. xylostella*^13^, which was the first report of miRNA-mediated chlorantraniliprole resistance in insects.

MiRNA is a type of endogenous, small non-coding RNA that plays important regulatory roles by targeting mRNAs for cleavage or translational repression. These small RNAs are usually 18–25 nt in length, and their precursors, which usually fold into stem-loop structures, are processed by Dicer endonuclease into two mature miRNAs, one from the plus strand and the other from the minus strand (star miRNA or miRNA*); in most cases, the star miRNA is presumed to be degraded^14^. The mature miRNA is loaded into an RNA-induced silencing complex (RISC), and it then guides the RISC to its specific mRNA target, where the miRNA “seed sequence” (nucleotides 2–8 at the 5′end) binds to the 3′ untranslated regions (3′UTR) of target mRNA, resulting in the repression of mRNA translation in animals or mRNA degradation in plants^15^,^16^. Other research has reported that some miRNAs could also bind to the 5′UTR^17^,^18^ or the open reading frame^19^ to suppress the expression of their target mRNAs.

The first miRNA was discovered in *Caenorhabditis elegans* over two decades ago^20^. Since then, a large number of miRNAs have been identified in many types of eukaryotes and viruses using a variety of methods.

The first group of miRNAs in *P. xylostella* was reported in 2013, when a total of 235 miRNAs were identified from second instar larvae under parasitic stress^21^. That same year, Liang *et al*. identified 462 miRNAs in all developmental stages of *P. xylostella*^22^. Although a number of miRNAs have been discovered in *P. xylostella*, a systematical identification of miRNAs associated with insecticide resistance in *P. xylostella* has not yet been conducted.

A laboratory-susceptible *P. xylostella* strain and two chlorantraniliprole-resistant strains were selected for this study. The global expression profiles of known and novel miRNAs were compared between the susceptible and two resistant strains, respectively, using high-throughput sequencing, and a batch of miRNAs associated with chlorantraniliprole resistance was obtained. We also predicted the targets of differentially expressed miRNAs by two different algorithms, and the functional annotation of the targets was also performed. These results will be helpful for further study of the role of miRNAs in the regulation of insecticide resistance in *P. xylostella*.

## Results

### Chlorantraniliprole resistance levels in CHS, CHR and ZZ

Chlorantraniliprole toxicity to different strains of *P. xylostella* is summarized in Table 1. The LC_50_ values of chlorantraniliprole for CHS, CHR and ZZ were 0.112 mg L^−1^, 5.097 mg L^−1^ and 4.681 mg L^−1^, respectively. That is, the resistance ratios for CHR and ZZ were 45.5- and 41.8- fold, respectively (Table 1).

### Sequencing of miRNAs from *P. xylostella*

**Figure 1:**
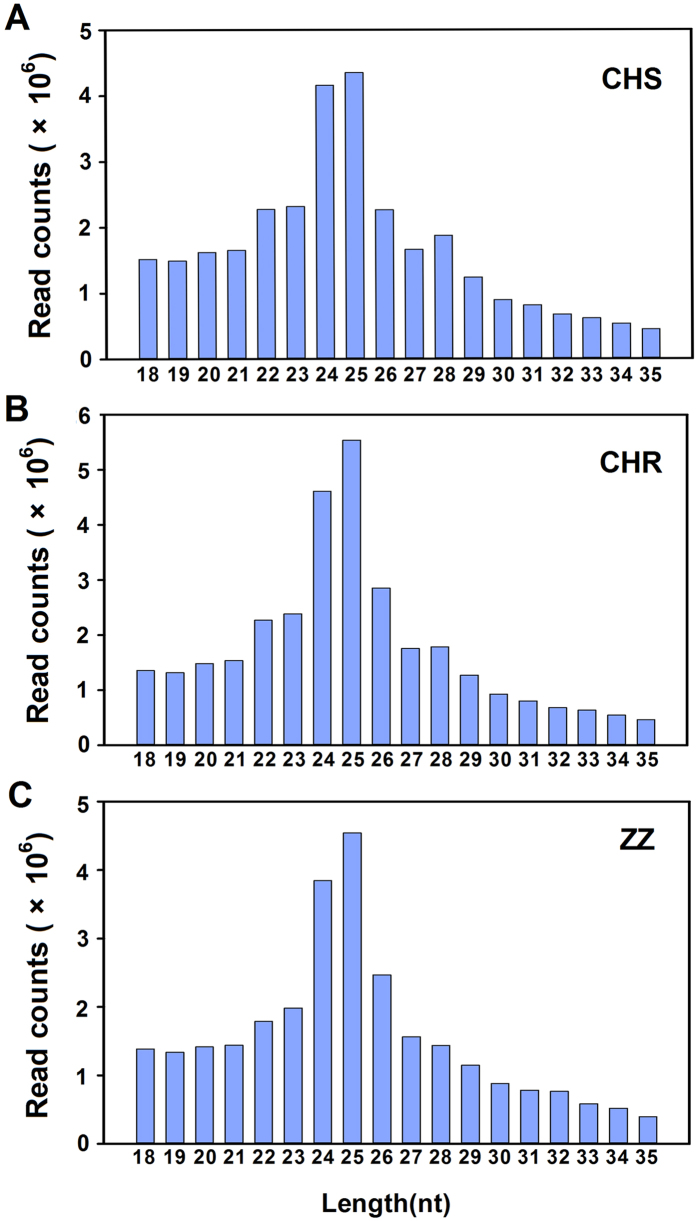
Size distribution of small RNAs in CHS, CHR and ZZ libraries.

Three small RNA (sRNA) libraries for CHS, CHR and ZZ were constructed, and 45,297,627, 47,566,639 and 40,906,561 raw reads were generated from the sRNA library for CHS, CHR and ZZ, respectively. The low-quality sequences, reads without a 3′ adaptor and reads that were less than 18 nt were eliminated; subsequently, 31,952,995, 33,561,726, and 29,46,332 clean reads were respectively obtained for the three strains and used for further analysis (Table 2). The length distributions are displayed in Fig. 1. The three libraries shared a similar distribution pattern, with 25 nt sRNAs being the most abundant, followed by 24, 26, 23 and 22 nt (Fig. 1).

### Identification of known miRNAs

Before this research, 235 miRNAs and 462 miRNAs in *P. xylostella* had already been identified by Etebari *et al*.^21^ and Liang *et al*.^22^, respectively, in 2013. However, a number of same or similar miRNAs in these two publications were named differently. Therefore, we collated all the miRNAs reported in these two references first, and then all clean sequences generated from this study were mapped against the resulting miRNAs. As a result, 199 known mature miRNA were obtained. Then the pre-miRNA sequences of these mature miRNAs were aligned to those reported by Etebari *et al*.^21^ and Liang *et al*.^22^ and mapped to the latest version of *P. xylostella* genome, and 121 confident pre-miRNA sequences were conformed, which produced 172 of 199 identified mature miRNAs (Table S1). However, the pre-miRNA sequences of the rest 27 conserved miRNAs were not detected in the current *P. xylostella* genome. Considering the incomplete assembly of this DBM genome version, these 27 conserved miRNAs were also used for further analysis (Table S2).

### Identification of novel miRNAs

After removal of the identified known miRNA sequences mentioned above, the rest of the clean sequences were processed to remove any non-coding RNAs, protein-coding RNA fragments and repeated sequences. Finally, 20,801,228 (CHS), 22,526, 217 (CHR) and 19,823,507 (ZZ) unannotated clean sequences were obtained and used to predict novel miRNAs (Table 2).

**Figure 2:**
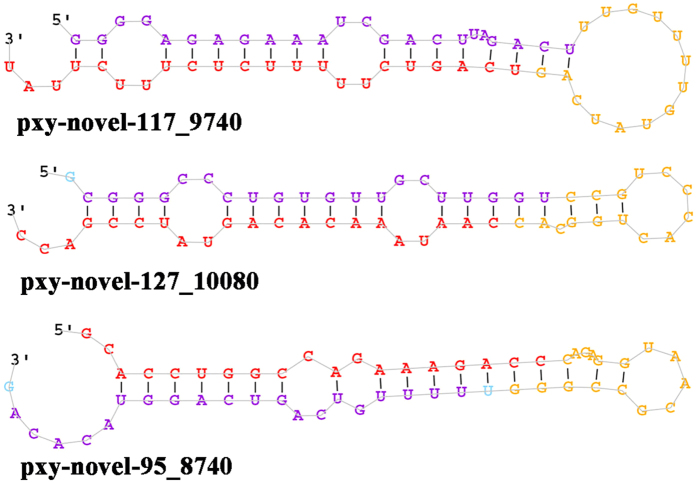
Predicted secondary structure of three selected novel miRNAs. The entire sequence represents pre-miRNAs, the red represents mature miRNA, and the purple represents miRNA*.

According to the unannotated sequences, miRNA precursor sequences and structures were predicted and identified using miRDeep2 (a probabilistic algorithm based on the miRNA biogenesis model)^23^, and a total of 51 potential novel miRNAs were initially predicted from the three libraries (Table S3). RNAfold was used to confirm the structures of the predicted miRNAs^24^. After keeping only novel miRNAs with a rand fold *P*-value ≤ 0.05 and a miRDeep2 score ≥3, 30 potential novel microRNAs were retained and used for expression analysis, and 24 were determined to have complementary star miRNA sequences (most of the star miRNAs have a low copy number). The length of the novel miRNAs ranged from 18 to 25 nt. Novel miRNAs were named based on their positions in the *P. xylostella* genome (Table S3). Secondary structures for some potential miRNA precursors with high miRDeep2 scores are shown in Fig. 2.

### The most abundant miRNAs in *P. xylostella*

MiR-Bantam, miR-10 and miR-281 were the three most abundant miRNAs in each of the three libraries. These three miRNAs were also highly expressed in *P. xylostella* second instar larvae reported by Etebari *et al*.^21^. Ten of the most highly expressed miRNAs are listed in Table 3, and a total of 20 miRNAs had high expression with a mean count number >10,000, including 2 novel miRNAs, pxy-novel-117_9740 and pxy-novel-95_8740 (Tables S1, S2, S3).

### Analysis of differentially expressed miRNAs

**Figure 3:**
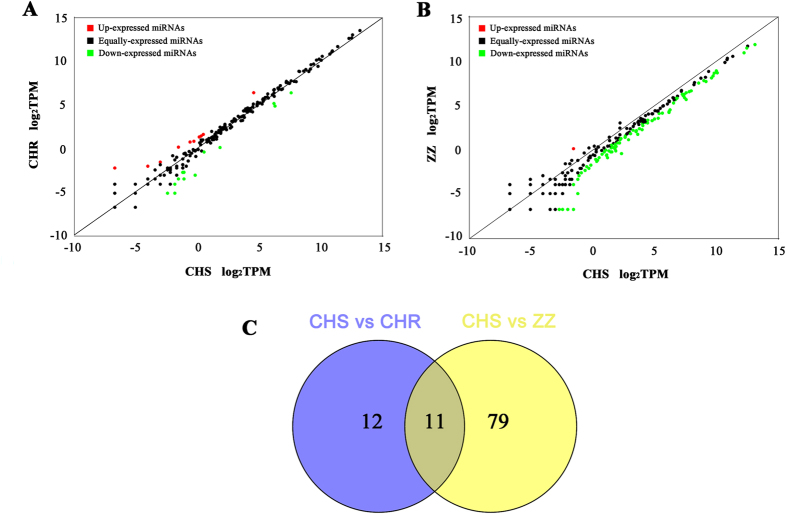
Differentially expressed miRNAs identified among CHS, CHR and ZZ. (**A**) Scatter plot of differentially expressed miRNAs between CHS and CHR; (**B**) Scatter plot of differentially expressed miRNAs between CHS and ZZ. Each point represents a miRNA. Red points indicate a fold change >1. Black points indicate −1< fold change <1. Green points indicate a fold change <−1; (**C**) Number of differentially expressed miRNAs among CHS, CHR and ZZ.

To systematically identify chlorantraniliprole resistance associated miRNAs, a differential expression analysis was performed among the three strains using the sequencing results. In total, 20 known and 3 novel miRNAs were identified as differentially expressed between CHR and CHS; 13 were significantly down-regulated, and 10 were significantly up-regulated (Table S4, Fig. 3A). In addition, 80 known and 10 novel miRNAs were differentially expressed between ZZ and CHS, 89 were significantly down-regulated, and only 1 were up-regulated (Table S5, Fig. 3B). Compared to CHS, 9 known and 2 novel miRNAs were found to be differentially expressed in both CHR and ZZ, 10 were down-regulated in both resistant strains, except pxy-miR-8491-5p, which was up-regulated (Fig. 3C, Table 4). Overall, most of the differentially expressed miRNAs were down-regulated in the resistant strains.

### Target prediction and annotation of differentially expressed miRNAs

Usually, miRNA functions by binding to its target mRNAs, therefore annotating the potential targets of differentially expressed miRNAs, and is very important in defining their roles in chlorantraniliprole resistance.

**Figure 4:**
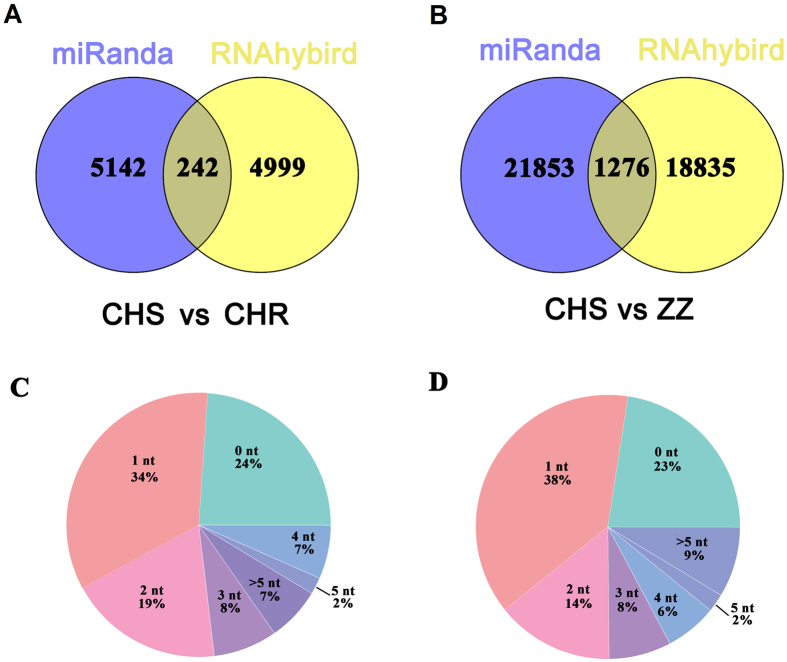
Identification of potential target genes for differentially expressed miRNAs among CHS, CHR and ZZ using miRanda and RNAhybrid. (**A**) Numbers of predicted target genes for the differentially expressed miRNAs between CHS and CHR using miRanda and RNAhybrid; (**B**) Numbers of predicted target genes for the differentially expressed miRNAs between CHS and ZZ using miRanda and RNAhybrid; (**C**) Binding site position variation in the two algorithms for the differentially expressed miRNAs between CHS and CHR; (**D**) Binding site position variation in the two algorithms for the differentially expressed miRNAs between CHS and ZZ.

For 23 significantly differentially expressed miRNAs between CHS and CHR, a total of 5,384 targets were predicted using miRanda, and 5,241 targets were identified using RNAHybird (Table S6, Fig. 4A). Similarly, for 90 significantly differentially expressed miRNAs between CHS and ZZ, 23,129 and 20,111 targets were predicted with miRanda and RNAHybird, respectively (Table S7, Fig. 4B).

To make the prediction results more reliable, the miRNA targets predicted by both miRanda and RNAhybrid were considered the final target genes. Finally, 242 miRNA-mRNA pairs between CHS and CHR (Table S8, Fig. 4A), and 1,276 miRNA-mRNA pairs between CHS and ZZ were supported by both algorithms (Table S9, Fig. 4B). Furthermore, all of the binding sites predicted by miRanda and RNAhybrid between miRNAs and their mRNA targets were counted. For different expressed miRNAs and their potential target mRNAs between CHS and CHR, 24% of the binding sites predicted by the two algorithms were almost identical (0 nt difference), and only 7% showed shifts more than 5 nt (Fig. 4C). Similarly, for different expressed miRNAs and their potential target mRNAs between CHS and ZZ, 23% of the binding sites predicted by the two algorithms were almost identical, and 9% showed shifts more than 5 nt (Fig. 4D).

A set of miRNAs were found to target several families of important genes that are often involved in insecticide resistance, such as cytochrome P450^25^,^26^,^27^,^28^, esterase^29^, GSTs^30^, ABC transporter family protein^31^,^32^, cuticle protein^33^, glutamate-gated chloride channel^34^,^35^ and superoxide dismutase (SOD)^36^. The target genes of some selected miRNAs are listed in Table 5. The final predicted targets for the differentially expressed miRNAs were used for qRT-PCR analysis, and their GO annotations and KEGG pathway mapping results are listed in Table S8 and Table S9, respectively.

### Quantitative RT-PCR validation of differentially expressed miRNAs and their potential targets

To validate the expression profiles of the differentially expressed miRNAs identified from the small RNA sequencing, a number of miRNAs were randomly selected for quantitative RT-PCR (qRT-PCR) assays.

**Figure 5:**
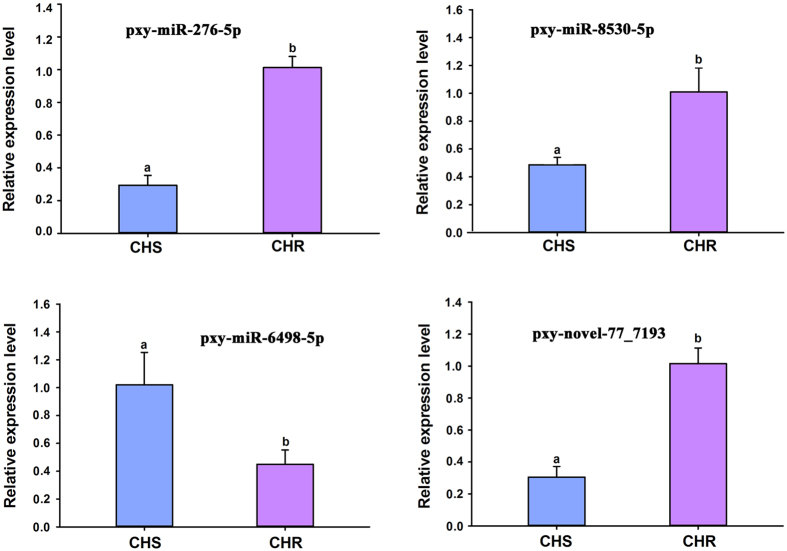
qRT-PCR validation of significantly differentially expressed miRNAs between CHS and CHR. Different lowercase letters (a and b) represent significant differences by t-test (*P* < 0.05). The same applies below.

**Figure 6:**
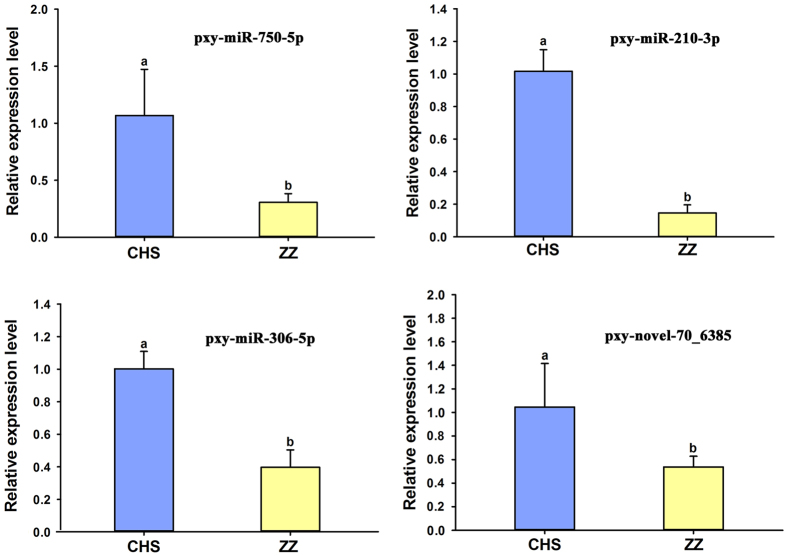
qRT-PCR validation of significantly differentially expressed miRNAs between CHS and ZZ.

**Figure 7:**
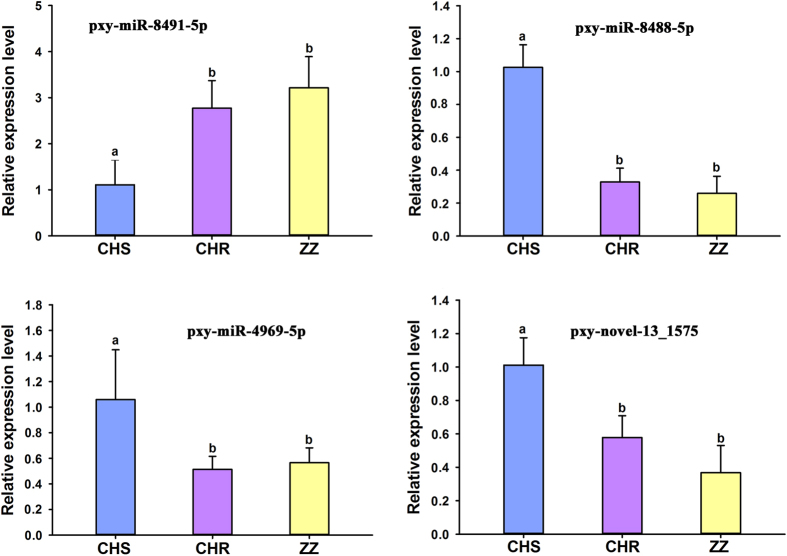
qRT-PCR validation of common differentially expressed miRNAs in CHR and ZZ compared to CHS.

Initially, 4 miRNAs (pxy-miR-276-5p, pxy-miR-6498-5p, pxy-miR-8530-5p and pxy-novel-77_7193) differentially expressed only between CHR and CHS (Fig. 5), 4 miRNAs (pxy-miR-750-5p, pxy-miR-210-3p, pxy-miR-306-5p and pxy-miR-965-3p) differentially expressed only between ZZ and CHS (Fig. 6), and 4 common differentially expressed miRNAs (pxy-miR-8491-5p, pxy-miR-4969-5p, pxy-mir-8488-5p and pxy-novel-13_1575) in both CHR and ZZ compared to CHS (Fig. 7) were used for qRT-PCR validation. The expression patterns of all selected miRNAs showed a similar trend between the results of sequencing and qRT-PCR.

**Figure 8:**
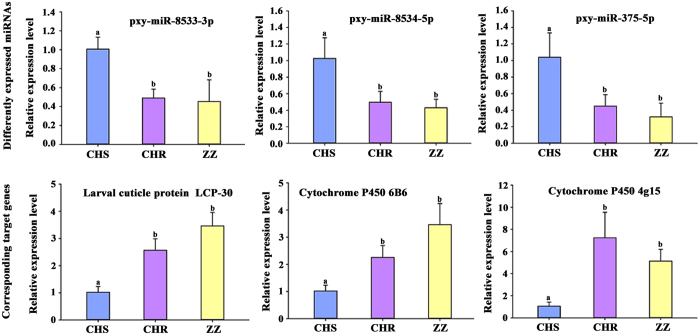
qRT-PCR analysis of significantly differentially expressed miRNAs and their potential targets.

To analyze the correlation between the expression levels of miRNAs and their potential targets, the relative expression of three miRNAs (pxy-miR-8533-3p, pxy-miR-8534-5p and pxy-miR-375-5p) down-regulated in both CHR and ZZ and their corresponding targets, including larval cuticle protein LCP-30, cytochrome P450 6B6 and cytochrome P450 4G15, were verified through qRT-PCR. The expression of the 3 selected miRNAs were all down-regulated, which shared a similar trend with the sequencing results, while the expression of their corresponding targets were all up-regulated (Fig. 8). All 3 selected miRNAs showed a significant negative correlation with their targets.

## Discussion

In recent years, an increasing number of papers on insect miRNA have been published^37^. A total of 3,824 mature miRNAs belonging to 26 species of insects have already been deposited in miRBase. MiRNA plays very important roles in the growth and development of insects, such as the reproductive process^38^ germ cell development^39^ neurogenesis^40^,^41^, wing development^42^,^43^,^44^, phenotypic plasticity^45^ and muscle growth^46^,^47^,^48^. More recently, miRNAs were also found to be involved in insecticide resistance^49^,^50^,^51^.

To systematically identify miRNAs associated with chlorantraniliprole resistance in *P. xylostella*, small RNAs from a susceptible strain (CHS) and two resistant strains (CHR, ZZ) were sequenced using Illumina sequencing technology in this study. The differentially expressed miRNAs among CHS, CHR and ZZ were analyzed, and their target genes were also predicted. A total of 229 miRNAs were identified in the three libraries, of which 199 miRNAs had already been reported in *P. xylostella* before, and 30 novel miRNAs were predicted using miRdeep2 for the first time.

Some highly conserved miRNAs, such as miR-let-7, miR-8, miR-9, miR-184, miR-278 and miR-bantam, which play essential roles in many types of insects^37^, were also discovered with high expression in the three *P. xylostella* strains, implying important regulatory roles in *P. xylostella*.

Twenty-three miRNAs were identified to be differentially expressed between CHR and CHS. Because CHR was established from CHS by successive selection with chlorantraniliprole (i.e., they have same genetic background) and have been reared under same laboratory conditions with CHS, the 23 differentially expressed miRNAs are likely to be associated with chlorantraniliprole resistance. Between ZZ and CHS, 90 differentially expressed miRNAs were identified. The ZZ strain was a field strain, and it had developed high levels of resistance to several other commonly used insecticides, such as beta-cypermethrin, abamectin, spinosad and indoxacarb (unpublished data from a local plant protection station), in addition to chlorantraniliprole. Each of these insecticides kills *P. xylostella* with distinctive modes of action. Therefore, the 90 differentially expressed miRNAs likely result from of the comprehensive effects of these different insecticides as well as other environmental factors.

When the differentially expressed 23 and 90 miRNAs were put together, we found that 11 of them overlapped. The overlapped miRNAs are likely to be involved in chlorantraniliprole resistance because they were differentially expressed in both laboratory-selected and field-collected resistant strains. However, due to the complex insecticide resistance mechanisms in the ZZ strain, the 11 miRNAs may also reveal common resistant mechanisms to other insecticides. In fact, some of them have been reported to be associated with several insecticides in different insects. For example, Hong *et al*. identified miR-375 and 27 other differentially expressed miRNAs between deltamethrin-susceptible and resistant *Culex pipiens* strains^49^. In this study, we found that an analog of miR-375 was differentially expressed between chlorantraniliprole susceptible and the two resistant *P. xylostella* strains, though with low abundance. These results, together with one P450 mRNA, imply that miR-375 has a high possibility of involvement in the regulation of insecticide resistance. The unique differentially expressed miRNAs in the CHR strain may reveal a unique mechanism of resistance to chlorantraniliprole; therefore, we should pay more attention to these 10 miRNAs in our follow-up work. The unique differentially expressed miRNAs in the ZZ strain, especially those that have different expression profiles compared with CHR, are more likely to be involved in other insecticide resistance and not chlorantraniliprole resistance. Some of them have been reported to be associated with deltamethrin, Cry1Ab or fenpropathrin resistance, such as miR-210, miR-965, miR-981, miR-1, miR-306 and miR-281^49^,^50^,^51^.

Because experimental validation of miRNA targets is still a major challenge, *in silico* prediction is widely used for the identification of potential miRNA targets^52^. In the current study, the target genes of the differentially expressed miRNAs among CHS, CHR and ZZ were predicted using two different algorithms, miRanda and RNAhybrid. Only the target genes supported by both algorithms were retained, and thus, the predicted results were relatively more credible. Although we did not find a ryanodine receptor in the predicted results, many other insecticide resistance associated genes were discovered, such as P450, esterase, GSTs, ABC transporter family protein, cuticle protein and SOD genes, of which several cytochrome P450, multidrug resistance-associated protein 4 (ABCC4) and SOD genes have already been identified to be differentially expressed among the susceptible strain and 3 different chlorantraniliprole-resistant *P. xylostella* strains^36^. Esterase FE4^29^, GSTT1^30^ and larval cuticle protein LCP-30 genes^33^ were also confirmed to be involved in resistance to other insecticides. In addition, some other metabolic enzymes were predicted to be associated with the differently expressed miRNAs, but there was no evidence for their involvement in insecticide resistance yet. Interestingly, several of them were discovered to be involved in detoxification process in mammal, such as UDP-glucuronosyltransferase 2A1^53^.

In this study, a number of miRNAs were speculated to be involved in chlorantraniliprole resistance in *P. xylostella* based only on their differential expression profiles and their predicted target genes. To further reveal the roles of these miRNAs in chlorantraniliprole resistance, gain- and loss-of-function experiments should be carried out *in vivo* and could include, for example, the up- or down-regulation of the expression of a miRNA by injecting its synthetic mimics or inhibitors and suppressing the expression of its target mRNAs by RNA interference (RNAi). This should be followed by the evaluation of resistance levels in treated larvae using bioassays.

## Conclusion

This paper presents the first study to systematically screen miRNAs associated with chlorantraniliprole resistance in *P. xylostella*. In this study, we identified 199 known miRNAs and 30 novel miRNAs in three DBM strains. A set of differentially expressed miRNAs among CHS, CHR and ZZ were obtained and considered highly likely to be associated with chlorantraniliprole resistance. The potential targets of the differentially expressed miRNAs were also predicted, and many of the target genes were related to detoxification processes. These results may guide us in further investigating the mechanisms of miRNA-regulated chlorantraniliprole resistance and may provide novel insights into resistance management in *P. xylostella*.

## Materials and Methods

### Insects

The laboratory susceptible DBM strain (CHS) was collected in the vegetable fields of Beijing and reared in our laboratory without exposure to any insecticide for more than 10 years. The chlorantraniliprole-resistant strain (CHR) was derived from the CHS strain by successive selection with chlorantraniliprole for more than 60 generations, and the field-resistant strain (ZZ) was collected in the vegetable fields of Zhangzhou, Fujian province, in southeastern China in 2015. All stages of *P. xylostella* were maintained at 27 ± 1 °C, an RH of 50–60% and a photoperiod of 16 h light/8 h dark on radish seedlings (*Raphanus sativus* L.). *P. xylostella* adults were provided with 10% (W/V) honey solution and allowed to mate and oviposit on the radish seedlings.

### Bioassay

The Leaf-dip method^54^ was used in this study. Cabbage leaves were dipped in the required chlorantraniliprole concentrations for 10–15 s and then allowed to air dry in the shade. A 0.1% (v/v) Triton X-100 water solution was used as a control. Approximately 20–25 third instar larvae were transferred onto each leaf, and three replications were used for each concentration. The mortality was assessed after four days of treatment. LC_50_ values were calculated using POLO-Plus 2.0 software (LeOra Software Inc., Berkeley, CA).

### Small RNA library construction and sequencing

RNA samples were prepared from the three DBM strains (each sample containing 30–50 third-instar *P. xylostella* larvae). Trizol Reagent was used to isolate the total RNA from each sample according to the manufacturer’s instructions (Invitrogen, Carlsbad, CA, USA). RNA degradation and contamination were assessed on 1% agarose gels, and RNA concentration was measured using the Nano Drop 2000 (Thermo, Wilmington, USA).

Next, 1 μg of total RNA were ligated sequentially with 3′and 5′ adaptors, and RT-PCR was performed using the TruSeq™ SmallRNA Sample Prep Kits (Illumina) for 15 cycles. The resulting ligation PCR products were isolated from a 6% TBE PAGE gel and sequenced using a HiseqXTEN sequencer (Illumina). Small RNA sequencing and bioinformatics analyses were conducted at the OE Biotechnology Company (Shanghai, China).

### Analysis of sequencing data

Data files from each of the three libraries were used for analysis. The raw data of this study were deposited in the NCBI Short Read Archive (SRX1968414, SRX1968415 and SRX1952874). Clean reads were screened from the raw data after processing out low-quality reads, adapters, and sequences of fewer than 18 nucleotides. Clean reads were mapped to the *P. xylostella* genome^55^ (version 2, http://iae.fafu.edu.cn/DBM/index.php) to analyze the distribution using bowtie software^56^.

All *P. xylostella* miRNAs reported by Etebari *et al*.^21^ and Liang *et al*.^22^ were collated and re-annotated first, then clean sequences generated in this research were used to search against the resulting miRNAs. All miRNAs identified in this step were considered known miRNAs.

All remaining clean sequences were subjected to the Rfam database to remove known noncoding RNA families, including rRNA, scRNA, snoRNA, snRNA and tRNA (http://www.sanger.ac.uk/Software/Rfam/ftp.shtml), and were then searched against known genes of *P. xylostella* to discard degraded fragments; the search was concluded in RepBase to remove repeated sequences (http://www.girinst.org). Unannotated clean sequences that did not match any of the above databases were further used to analyze and predict novel miRNAs using miRDeep2 software. Both a false positive rate (FPR) and true positive rate (TPR) were used to assess the predicted results, and RNAfold was used to confirm the structures of the predicted miRNAs.

### Differential Expression Analysis of miRNAs

The abundance of miRNAs identified in the three libraries was first normalized using the tags per million reads (TPM) method: TPM = (number of mapped reads for each miRNA/total number of mapped reads) ×10^6^. The log_2_ (TPM ratios) among the three libraries was calculated, and the *P*-value was calculated using the Audic Claverie statistic. The miRNAs with |log_2_ (TPM ratios)| ≥ 1 and *P*-value < 0.05 were regarded as differentially expressed among the three *P. xylostella* strains.

### Target prediction and annotation of differentially expressed miRNAs

MiRNA usually regulates gene expression through binding to the 3′ untranslated region (3′ UTR) of target mRNAs, so 3′ UTR annotation information was first extracted from the genome database of *P. xylostella* for target prediction. The potential target genes of differentially expressed miRNAs were predicted and analyzed using two different types of software, miRanda^57^ and RNAhybrid^58^,^59^. For each prediction method, high efficacy targets were selected by the following criteria: (1) miRanda: total score ≥ 140, total energy ≤ −25 kcal/mol; (2) RNAhybrid: *P*-value < 0.05, mfe ≤ −25 kcal/mol.

### Verification of differentially expressed miRNAs and their potential targets by quantitative real-time PCR

Quantitative real-time PCR was performed to experimentally validate the relative expression levels of the identified miRNAs and their potential targets. Total RNA was extracted from the same samples used for deep sequencing. The first-strand cDNA of mature miRNA and mRNA were synthesized using a miScript II RT kit (Qiagen, Germany) and a PrimeScript™ RT reagent Kit with gDNA Eraser (Perfect Real Time) (Takara Biotechnology, Dalian, China), respectively, following the manufacturer’s instructions. qRT-PCR analysis was carried out using SYBR Premix Ex Taq (Takara Biotechnology, Dalian, China). Each reaction was performed in an ABI 7500 Real Time PCR system (Applied Biosystems) with three biological replicates. The expression levels for miRNA and mRNA were normalized to U6 snRNA and ribosomal protein L32 mRNA, respectively. The relative expression levels of the miRNAs and targets were calculated using the 2^–ΔΔCt^ method^60^. All primers used in this study are listed in Table S10.

## Additional Information

**How to cite this article**: Zhu, B. *et al*. Global identification of microRNAs associated with chlorantraniliprole resistance in diamondback moth *Plutella xylostella* (L.). *Sci. Rep.* **7**, 40713; doi: 10.1038/srep40713 (2017).

**Publisher's note:** Springer Nature remains neutral with regard to jurisdictional claims in published maps and institutional affiliations.

## Supplementary Material

Supplementary Information

Supplementary Dataset 1

Supplementary Dataset 2

Supplementary Dataset 3

Supplementary Dataset 4

Supplementary Dataset 5

Supplementary Dataset 6

Supplementary Dataset 7

Supplementary Dataset 8

Supplementary Dataset 9

Supplementary Dataset 10

## Figures and Tables

**Table 1:** Toxicity of chlorantraniliprole to different strains of Plutella xylostella.

Strain	Number^a^	LC50 (mg L^−1^) (95% CL^b^)	Slope ± SE	χ^2^ (df)^c^	RR at LC50^d^
CHS	351	0.112 (0.099–0.125)	3.172 ± 0.348	7.292 (13)	-
CHR	357	5.097 (4.586–5.573)	4.292 ± 0.484	7.958 (13)	45.51
ZZ	360	4.681 (4.238–5.107)	4.345 ± 0.376	8.817 (13)	41.79

^a^Number of larvae assayed; ^b^Confidence limits; ^c^Chi-square value (χ2) and degrees of freedom (df) as calculated by PoloPlus; ^d^RR: Resistance ratio = LC50 of CHR or ZZ/LC50 of CHS.

**Table 2:** Categorization and abundance of sRNA reads from CHS, CHR and ZZ.

Data Processing/Strain	Reads all			Reads unique		
	CHS	CHR	ZZ	CHS	CHR	ZZ
Raw reads	45297627	47566639	40906561	5066237	5421221	4643140
Clean reads	31952995	33561726	29468332	4076241	4431220	3484682
Mapped to *P. xylostella* miRNAs reported by Etebari *et al*. or Liang *et al*. in 2013^22^	1400099 (4.38%)	1448802 (4.31%)	621992 (2.20%)	1595 (0.04%)	1602 (0.03%)	1350 (0.04%)
Mapped to other RNAs (RFam: rRNA, tRNA, snRNA, snoRNA and others)	7781970 (24.35%)	7883299 (23.49%)	7968865 (27.04%)	314018 (7.70%)	313629 (7.08%)	311243 (8.93%)
Mapped to known *P. xylostella* genes	1427410 (4.47%)	1199346 (3.57%)	552586 (1.88%)	483528 (11.86%)	434144 (9.80%)	222222 (6.38%)
Mapped to Repbase	542288 (1.70%)	504062 (1.50%)	501382 (1.70%)	23230 (0.57%)	20743 (0.47%)	20311 (0.58%)
Unannotated Sequences	20801228 (65.10%)	22526217 (67.12%)	19823507 (67.27%)	3253872 (79.83%)	3661102 (82.62%)	2929556 (84.07%)

**Table 3:** 10 of most highly expressed miRNAs in *P. xylostella* small RNA libraries.

MiRNA	Absolute read counts			Mature sequence
	CHS	CHR	ZZ	
pxy-bantam-3p	268877	285557	99336	UGAGAUCAUUGUGAAAGCUGAU
pxy-miR-10-5p	177805	204671	88001	UACCCUGUAGAUCCGAAUUUGU
pxy-miR-281-5p	170250	160753	76068	AAGAGAGCUAUCCGUCGACAGUA
pxy-miR-8-3p	145792	155955	52901	UAAUACUGUCAGGUAAAGAUGUC
pxy-miR-31-5p	78039	82910	40113	AGGCAAGAUGUCGGCAUAGCUGA
pxy-miR-184-3p	57775	59141	34587	UGGACGGAGAACUGAUAAGGGC
pxy-miR-263a-5p	56688	55596	31236	AAUGGCACUGAAAGAAUUCACGGG
pxy-miR-6094-3p	50483	46670	25105	UAUUCGAGACCUCUGCUGAUCCU
pxy-miR-279b-3p	32917	39015	11240	UGACUAGAUUUUCACUCAUCCUA
pxy-miR-9b-5p	31184	29007	13145	UCUUUGGUAUUCUAGCUGUAG

**Table 4:** Common differentially expressed miRNAs in CHR and ZZ compared to CHS.

MiRNA	CHR/CHS Log_2_ ratio	ZZ/CHS Log_2_ ratio	UP_ down
pxy-mir-8487-3p	−1.20	−1.97	Down
pxy-miR-8488-5p	−2.66	−4.23	Down
pxy-miR-8491-5p	1.60	1.66	Up
pxy-miR-8533-3p	−1.01	−1.41	Down
pxy-miR-8534-5p	−1.66	−1.79	Down
pxy-miR-375-5p	−1.95	−2.34	Down
pxy-miR-4969-5p	−1.57	−1.76	Down
pxy-miR-625_85053-3p	−1.40	−1.91	Down
pxy-miR-64_964078-5p	−1.78	−1.72	Down
pxy-novel-13_1575	−1.56	−3.69	Down
pxy-novel-293_16368-5p	−2.39	−1.79	Down

**Table 5:** Potential target genes of miRNAs that have already been identified to be involved in insecticide resistance.

MiRNA	Target id	Annotation	Related insecticide	References
pxy-miR-8533-3p	Px003252	Larval cuticle protein LCP-30	Thiamethoxam	Pan *et al*.^33^
pxy-miR-100-5p	Px013707	Cytochrome P450 4V2	Phoxim; Chlorantraniliprole	Gu *et al*.^26^; Lin *et al*.^36^
	Px012816	Glutamate-gated chloride channel	Fipronil; Avermectins	Ikeda *et al*.^34^; Bloomquist *et al*.^35^
pxy-miR-8534-5p	Px005902	Cytochrome P450 6B6	Deltamethrin; Chlorantraniliprole	Zhou *et al*.^25^; Lin *et al*.^36^
pxy-let-7-3p	Px011036	Glutathione S-transferase T1	Chlorpyrifos	Qin *et al*.^30^
pxy-miR-11-3p	Px017041	Superoxide dismutase [Cu-Zn]	Chlorantraniliprole; Chlorantraniliprole	Lin *et al*.^36^
pxy-miR-2525-3p	Px005902	Cytochrome P450 6B6	Deltamethrin	Zhou *et al*.^25^
pxy-miR-275-5p	Px014440	Cytochrome P450 9e2	Phosphine	Oppert *et al*.^27^
	Px011552	Multidrug resistance-associated protein 4	Pyrethroid; DDT; Lindane; Chlorantraniliprole	Bariami *et al*.^31^; Lu *et al*.^32^; Lin *et al*.^36^
pxy-miR-1175-5p	Px004046	Esterase FE4	Naled	Hsu *et al*.^29^
pxy-miR-279c-3p	Px005900	Cytochrome P450 6B2	*Bacillus thuringiensis* BT	Muñoz *et al*.^28^
