# Supplementary material for: Global identification of microRNAs associated with chlorantraniliprole resistance in diamondback moth Plutella xylostella (L.)
Source: Sci Rep. 2017 Jan 18;7:40713. doi: 10.1038/srep40713 (PMC5241650; doi:10.1038/srep40713)
Supplement: Supplementary Information [file srep40713-s1.doc]

**Global identification of microRNAs associated with chlorantraniliprole resistance in diamondback moth *Plutella xylostella* (L.)**

Bin Zhu, Xiuxia Li, Ying Liu, Xiwu Gao, Pei Liang*

*Department of Entomology, China Agricultural University, Beijing, 100193, P. R. China*

**Supporting Information:**

**Table S1:** **Known miRNAs identified in this research and their pre-miRNA sequences.**

**Table S2: Conserved miRNAs identified in this research whose pre-miRNA sequence was not detected in the DBM genome Version 2.**

**Table S3: Novel miRNAs predicted in this research.**

**Table S4: Differentially expressed miRNAs between CHR and CHS.**

**Table S5: Differentially expressed miRNAs between ZZ and CHS.**

**Table S6: Target prediction of differentially expressed miRNAs between CHR and CHS by using miRanda and RNAhybrid.**

**Table S7: Target prediction of differentially expressed miRNAs between ZZ and CHS by using miRanda and RNAhybrid.**

**Table S8: Finally predicted targets of differentially expressed miRNAs between CHR and CHS supported by both the two algorithms, miRanda and RNAhybrid.**

**Table S9: Finally predicted targets of differentially expressed miRNAs between ZZ and CHS supported by both the two algorithms, miRanda and RNAhybrid.**

**Table S10: All primers used in this research.**
